# Seven oxidative stress-related genes predict the prognosis of hepatocellular carcinoma

**DOI:** 10.18632/aging.205330

**Published:** 2023-12-14

**Authors:** Chen Miao, Xiao He, Gang Chen, Ulf D. Kahlert, Chenchen Yao, Wenjie Shi, Dongming Su, Liang Hu, Zhihong Zhang

**Affiliations:** 1 Department of Pathology, The First Affiliated Hospital of Nanjing Medical University, Nanjing, China; 2 Molecular and Experimental Surgery, Clinic for General-, Visceral-, Vascular and Transplant Surgery, Faculty of Medicine and University Hospital Magdeburg, Otto-von-Guericke University Magdeburg, Magdeburg, Germany; 3 Department of Pathology, Nanjing Medical University, Nanjing, China; 4 Department of Pathology and Clinical Laboratory, Sir Run Run Hospital of Nanjing Medical University, Nanjing, China; 5 Neuroprotective Drug Discovery Key Laboratory of Nanjing Medical University, Department of Pharmacology, Nanjing Medical University, Nanjing, China

**Keywords:** hepatocellular carcinoma, Alpha-Enolase 1, N-myc downstream-regulated gene 1, nucleophosmin, Cox regression model

## Abstract

Predicting the prognosis of hepatocellular carcinoma (HCC) is a major medical challenge and of guiding significance for treatment. This study explored the actual relevance of RNA expression in predicting HCC prognosis. Cox's multiple regression was used to establish a risk score staging classification and to predict the HCC patients’ prognosis on the basis of data in the Cancer Genome Atlas (TCGA). We screened seven gene biomarkers related to the prognosis of HCC from the perspective of oxidative stress, including Alpha-Enolase 1(ENO1), N-myc downstream-regulated gene 1 (NDRG1), nucleophosmin (NPM1), metallothionein-3, H2A histone family member X, Thioredoxin reductase 1 (TXNRD1) and interleukin 33 (IL-33). Among them we measured the expression of ENO1, NGDP1, NPM1, TXNRD1 and IL-33 to investigate the reliability of the multi-index prediction. The first four markers’ expressions increased successively in the paracellular tissues, the hepatocellular carcinoma samples (from patients with better prognosis) and the hepatocellular carcinoma samples (from patients with poor prognosis), while IL-33 showed the opposite trend. The seven genes increased the sensitivity and specificity of the predictive model, resulting in a significant increase in overall confidence. Compared with the patients with higher-risk scores, the survival rates with lower-risk scores are significantly increased. Risk score is more accurate in predicting the prognosis HCC patients than other clinical factors. In conclusion, we use the Cox regression model to identify seven oxidative stress-related genes, investigate the reliability of the multi-index prediction, and develop a risk staging model for predicting the prognosis of HCC patients and guiding precise treatment strategy.

## INTRODUCTION

Liver cancer seriously endangers people’s health. It is conservatively estimated that 1 million people will be affected by liver cancer each year [1]. Hepatocellular carcinoma (HCC) is the predominant type of liver cancer and is associated with persistent inflammation and fibrosis [2]. Post-treatment recurrence is noted up to 60% of patients following partial liver resection, and about 15% of liver transplant patients [3]. In previous studies, Macrovascular invasion, Lymphovascular invasion and poor differentiation have been used to speculate the clinical outcomes of patients [4]. However, liver cancer is an extraordinarily heterogeneous malignant disease, we need to search for novel biomarkers to provide a more precise prognosis for doctors.

HCC is a heterogeneous malignancy with dismal prognosis. TERT, TP53 and CTNNB1 mutations are the most common mutations that affect the prognosis of HCC. However, these mutations are not fully present in most patients, and their clinical use is not yet widely accepted. To date, specific biomarkers are still needed to improve the prognosis of HCC. Compared with a single biomarker, polygenic markers can improve the specificity of the prognosis of tumor patients.

In the past few decades, considerable progress has been made in understanding the biomarkers and molecular characteristics of HCC [5]. The most studied prognostic parameters were tumor number, size, α-Feto protein (AFP) level, cell differentiation, MVI and ES grades, presence of satellite nodule, and pTNM stage [6]. However, existing prognostic staging systems still have many limitations in guiding treatment and prognosis. Recently, there has been increasing evidence that oxidative stress plays a crucial role in the development of liver cancer [7]. The occurrence of liver cancer is closely related to oxidative stress and inflammation [8]. Excessive and long-term inflammation and oxidative stress can cause irreversible damage and may lead to cirrhosis and carcinogenic transformation, while liver cancer is the inevitable result of the development of liver cirrhosis. Therefore, understanding the molecular mechanism of oxidative stress in hepatocellular carcinoma and its effect on prognosis will help to provide new therapeutic strategies for the treatment of HCC. Shen et al. investigated that Facilitates Chromatin Transcription complex was remarkably upregulated in HCC, which mediated oxidative stress to promote HCC progression [9]. Similarly, as a biomarker for oxidative stress, high expression of 8-Hydroxy-2-deoxyguanosine is associated with poor survival in HCC patients [10]. Recent findings have reported that oxidative stress promotes thyroid cancer development by upregulating protein Tyrosine Phosphatases expression [11]. Furthermore, the degradation of mitogen-activated protein kinase phosphatase-3 mediated by oxidative stress contributes to tumorigenicity of human ovarian cancer cells [12].

The above studies support that those biomarkers are useful tools for oxidative stress, and they do have potential in determining the stage of tumor progression. However, compared with single biomarker, polygenic markers can improve the specificity of tumor patient prognosis [13]. Paik et al. found that a 21-gene recurrence score predicts response to chemotherapy in breast cancer [14], indicating the superiority of polygenic markers in predicting prognosis.

Here, we used Cox multiple regression models to evaluate gene expression in HCC cases from The Cancer Genome Atlas (TCGA; http://www.tcga.org/). Compared with the patients with high-risk scores, the survival rates of the patients with low-risk scores are significantly higher. The findings were further validated with training and complete test datasets. Furthermore, we found that risk scores can be independent of other clinical variables, has a greater advantage in judging the prognosis of HCC. Combining the risk score with other clinical factors to form a nomogram to predict the 5-year and 10-year survival rates of HCC patients is more accurate and convenient.

## RESULTS

### Differential oxidative stress-related genes

**Figure 1:**
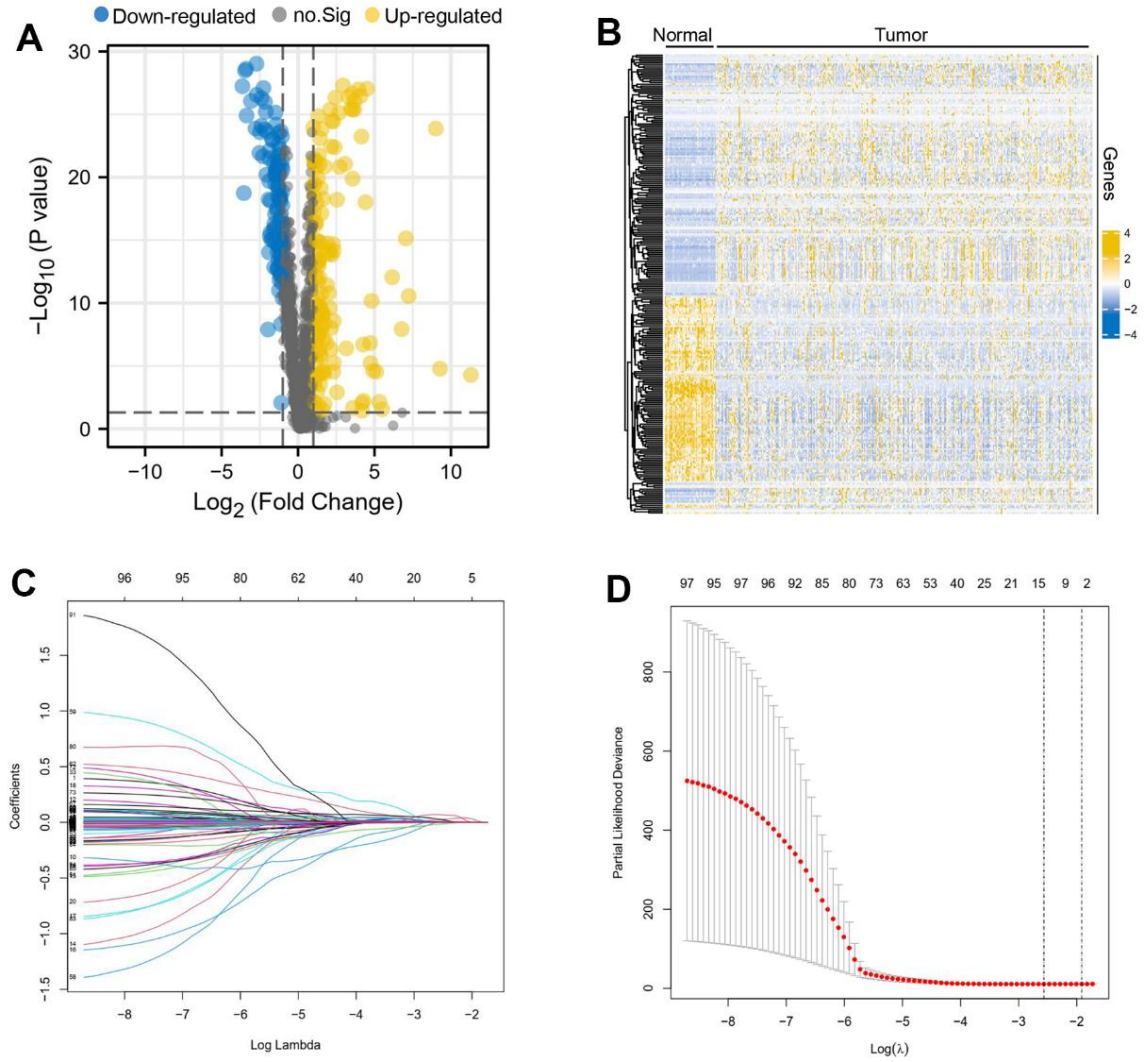
**Differential oxidative stress-related genes between liver cancer and normal tissues.** A volcano map (**A**) and heatmap (**B**) about differential oxidative stress-related genes was created, the down-regulated genes were expressed in blue, the up-regulated genes in yellow and the genes that did not differ between groups were represented in grey. Lambda obtained minimum value (**C**), model enrolled 11 important genes (**D**).

Analysis of differential gene expression between liver cancer and normal tissues suggested that a total of 254 genes were dysregulated, of which 145 were up-regulated and 109 were down-regulated (Supplementary Table 1). We visualised the differences and created a volcano map and heat map, in volcano map the down-regulated genes were expressed in blue, the up-regulated genes in yellow and the genes that did not differ between groups were represented in grey (Figure 1A, 1B). LASSO regression results demonstrate that Lambda obtained minimum value (Figure 1C), model enrolled 11 important genes (Figure 1D).

### Prognosis-related oxidative stress-related genes

Univariate Cox regression demonstrates 98 genes could affect patients’ outcome (Supplementary Table 2). When these 98 genes were entered into the LASSO regression model, the number of genes that met the Lambda minimum became 11, namely CHEK1, ENO1, EZH2, G6PD, H2AX, IL33, IRAK1, MT3, NDRG1, NPM1, and TXNRD1.

### Seven gene signatures of oxidative stress-related genes can predict patient prognosis

**Figure 2:**
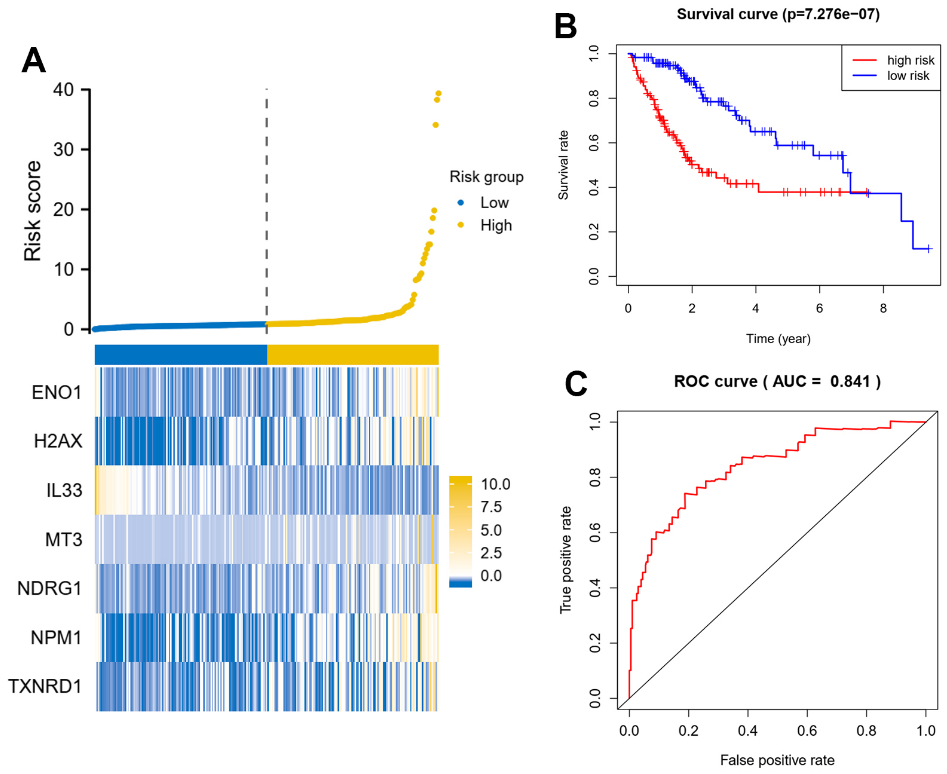
**Seven oxidative stress-related genes predict patient prognosis model in training dataset.** (**A**) A signature model for each of the 7 genes was constructed, and the risk score for each patient based on the model was calculated. X axis is number of patients. (**B**) Survival analysis about patients in the high-risk group and the low-risk group. (**C**) Analysis of the area under the ROC curve. The signature model based on Cox regression coefficients and gene expression, the riskscore = gene A expression* coefficients A+ gene B expression* coefficients B.

**Figure 3:**
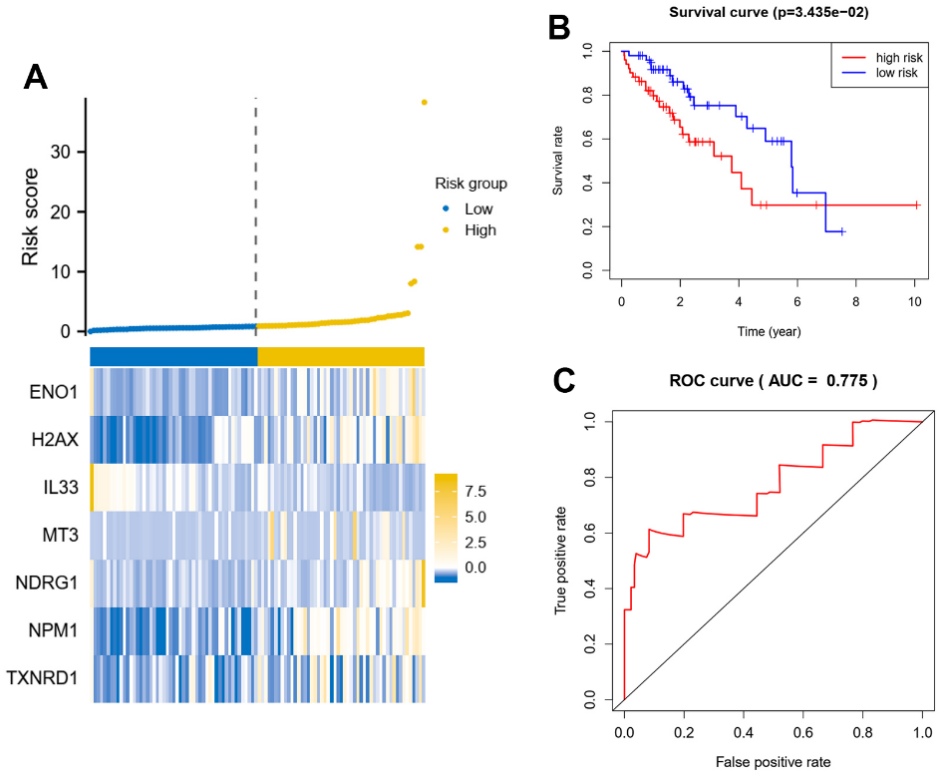
**Seven oxidative stress-related genes predict patient prognosis model in test dataset.** (**A**) A signature model for each of the 7 genes was constructed, and the risk score for each patient based on the model was calculated. X axis is number of patients. (**B**) Survival analysis about patients in the high-risk group and the low-risk group. (**C**) Analysis of the area under the ROC curve. The signature model based on Cox regression coefficients and gene expression, the riskscore = gene A expression* coefficients A+ gene B expression* coefficients B.

Multifactorial Cox regression of 11 prognostic genes resulted in seven independent risk genes affecting the prognosis of patients with hepatocellular carcinoma, namely ENO1, NDRG1, NPM1, H2AX, IL33, MT3 and TXNRD1 (Table 1). Based on the expression of the genes and the model coefficients, we constructed a signature model for each of the 7 genes and calculated the risk score for each patient based on the model (Figure 2A). Survival analysis revealed that patients in the high-risk group had a worse prognosis, compared to patients in the low-risk group, who had a significantly longer survival time (Figure 2B). In addition, the area under the ROC curve was equal to 0.841, suggesting that the model was highly discriminatory in terms of patient stratification (Figure 2C). This conclusion is also supported by the data from this validation set (Figure 3A–3C).

**Table 1:** Multi-Cox regression of 11 prognostic genes resulted in seven independent risk genes affecting the prognosis of patients with hepatocellular carcinoma.

**Gene**	**coef**	**HR**	**HR.95L**	**HR.95H**	**p-value**
ENO1	0.000568	1.000568	0.999953	1.001184	0.070251
NDRG1	0.00858	1.008616	1.002766	1.014501	0.003848
NPM1	0.003667	1.003673	0.999362	1.008003	0.095043
MT3	0.106454	1.112326	1.047154	1.181556	0.000549
TXNRD1	0.009974	1.010024	1.004602	1.015476	0.000282
H2AX	0.022586	1.022843	1.008564	1.037324	0.001639
IL33	-0.13473	0.87395	0.782905	0.975583	0.016378

### Seven oxidative stress-related genes detect early-stage liver cancer

**Figure 4:**
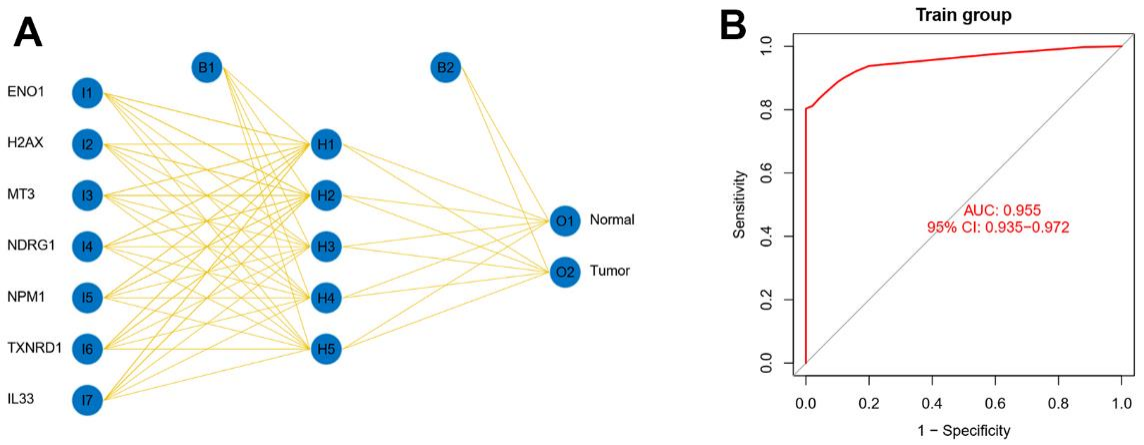
**Seven oxidative stress-related genes detect early-stage liver cancer.** (**A**) The model genes in the signature using the BP network were analyzed and the network was visualized. (**B**) The seven genes were used as a panel for early detection of liver cancer.

The oxidative stress-related gene signature of the seven genes was derived from a differential analysis of cancer and paracancer, so we hypothesized that it could not only predict the prognosis of patients but also have the potential to detect early-stage liver cancer, and therefore we further analyzed the model genes in the signature using the BP network and visualized the network (Figure 4A and Supplementary Table 3). The results suggested that these seven genes could be used as a panel for early detection of liver cancer with high early detection efficacy (ROC=0.955) (Figure 4B).

### ENO1, NDGR1, NPM1 and TXNRD1 are highly expressed in HCC tissues from patients with poor prognosis

**Figure 5:**
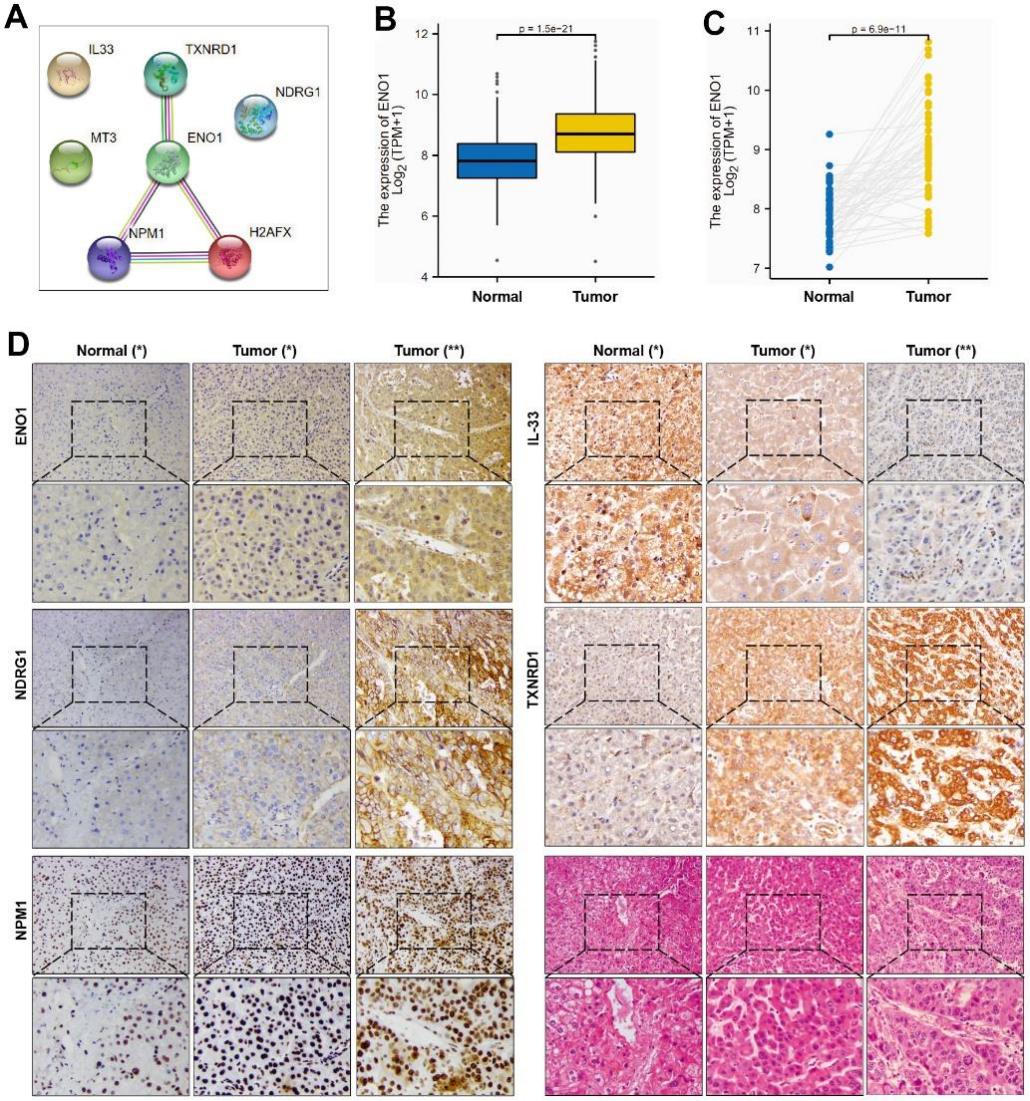
**ENO1, NDGR1 and NPM1 expression in HCC tissues.** (**A**) PPI network identifies ENO1 as hub gene of signature. (**B**, **C**) The expression of ENO1 gene in paracellular tissues and hepatocellular carcinoma tissues. (**D**) The protein expression of ENO1, NDGR1, NPM1, TXNRD1 and IL-33 in hepatocellular carcinoma tissues and paracellular tissues (n= 10 per group). Samples were stained with H&E staining to observe the structure of paracellular tissues and hepatocellular carcinoma tissues (n= 10 per group). Patients’ essential characteristics were shown in Supplementary Table 4. Normal (*): paracellular tissues; Tumor (*): hepatocellular carcinoma tissues, EDMONDSON Classification: II; Microvascular tumor thrombus: MVI grade 0; Tumor (**): hepatocellular carcinoma tissues, EDMONDSON Classification: III-IV; Microvascular tumor thrombus: MVI grade 1.

PPI network identifies ENO1 as hub gene of signature (Figure 5A). We examined the expression of ENO1 gene in hepatocellular carcinoma tissues at both transcriptomic levels and showed that ENO1 gene was significantly more highly expressed in both paired and unpaired hepatocellular carcinoma samples compared to paracellular tissues (Figure 5B, 5C). To further verify the effectiveness of ENO1, we performed the validation studies. As shown in Figure 5D, immunohistochemistry experiment was used to measure the expression of ENO1 in paracellular tissues and hepatocellular carcinoma sample. Compared with the paracellular tissues (Normal * group), the expression of ENO1 was increased in the hepatocellular carcinoma samples (Tumor * group). Furthermore, we found that ENO1 expression in the hepatocellular carcinoma samples from patients with poor prognosis (Tumor ** group) was higher than the samples from patients with better prognosis (Tumor * group). Additionally, we measured the expression of another four targets (NDRG1, NPM1, TXNRD1 and IL-33) to investigate the reliability of the multi-index prediction. As shown in Figure 5D, compared with the paracellular tissues, the expression of NDRG1, NPM1, TXNRD1 were both increased in the hepatocellular carcinoma samples. We also found that the expression of NDRG1, NPM1, TXNRD1 in the hepatocellular carcinoma samples from patients with poor prognosis were higher than the samples from patients with better prognosis. In contrast, the expression of IL-33 was decreased in the hepatocellular carcinoma samples. We also found that IL-33 expression in the hepatocellular carcinoma samples from patients with poor prognosis were lower than the samples from patients with better prognosis. Meanwhile, the samples were observed using haematoxylin and eosin (H&E) staining. As shown in Figure 5D, Normal (*): Normal liver morphology adjacent to cancer. Tumor (*): Organization type: trabecular type; Cell type: common type; EDMONDSON classification: II; Microvascular tumor thrombus: MVI grade 0. Tumor (**): Tissue type: trabecular type, pseudoadenoid type; Cell types: common, tumor giant cell type, hyaline cell type; EDMONDSON classification: III-IV; Microvascular tumor thrombus: MVI grade 1, the cells are heterotypic, the nucleus is large and divided. These data suggested that the oxidative stress-related polygenic biomarkers we screened were reliable and clinically significant in predicting prognosis.

### High expression of hub gene ENO1 suggests disease progression and correlates with tumor immune infiltration

**Figure 6:**
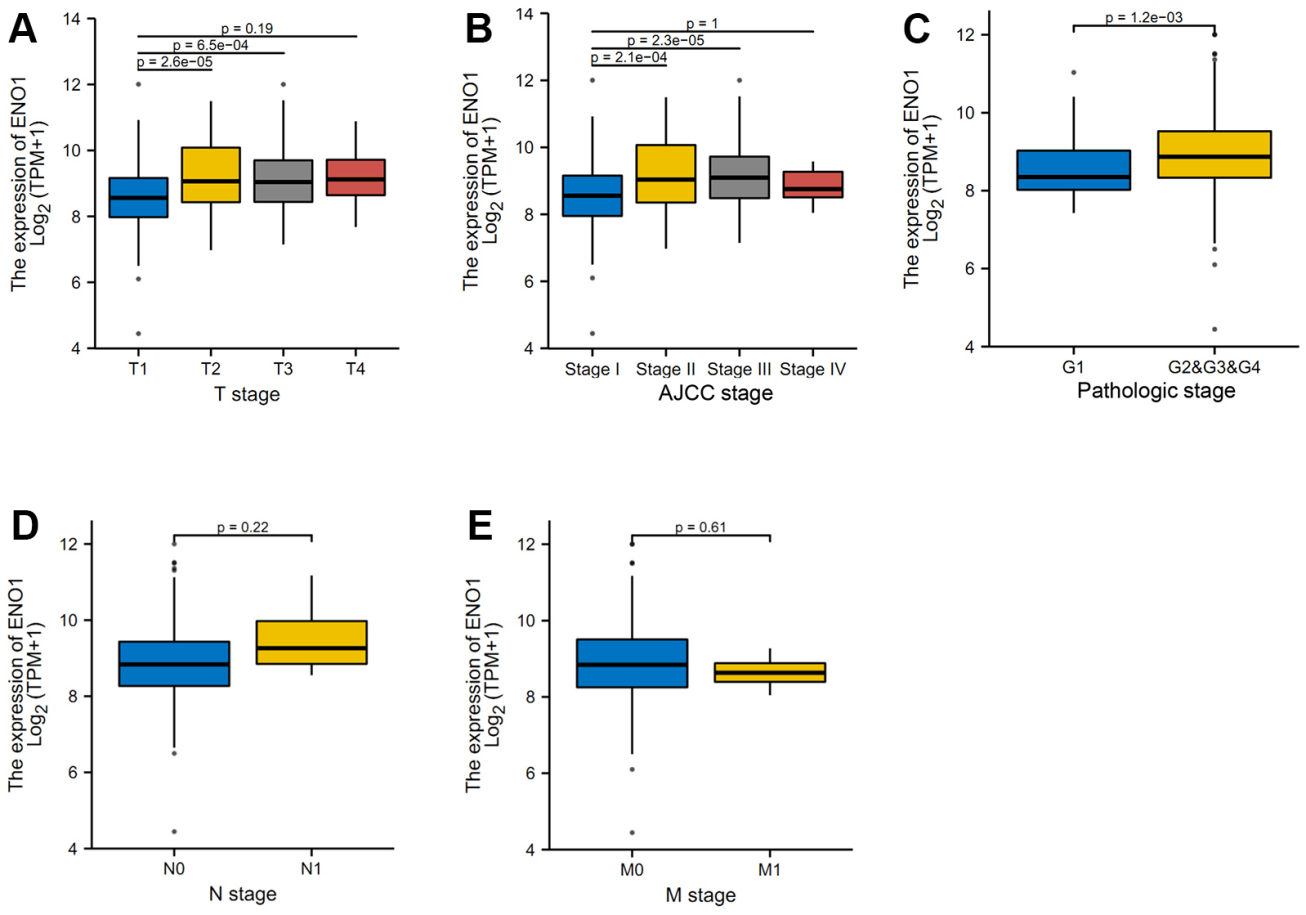
**High expression of hub gene ENO1 suggests disease progression.** (**A**) The clinical correlation analysis of ENO1 gene expression and tumor size and stage. The expression of ENO1 in different tumor stages (**B**), and in the pathological Grade stage (**C**). ENO1 was not associated with lymph node status (**D**) or distant metastatic status (**E**).

**Figure 7:**
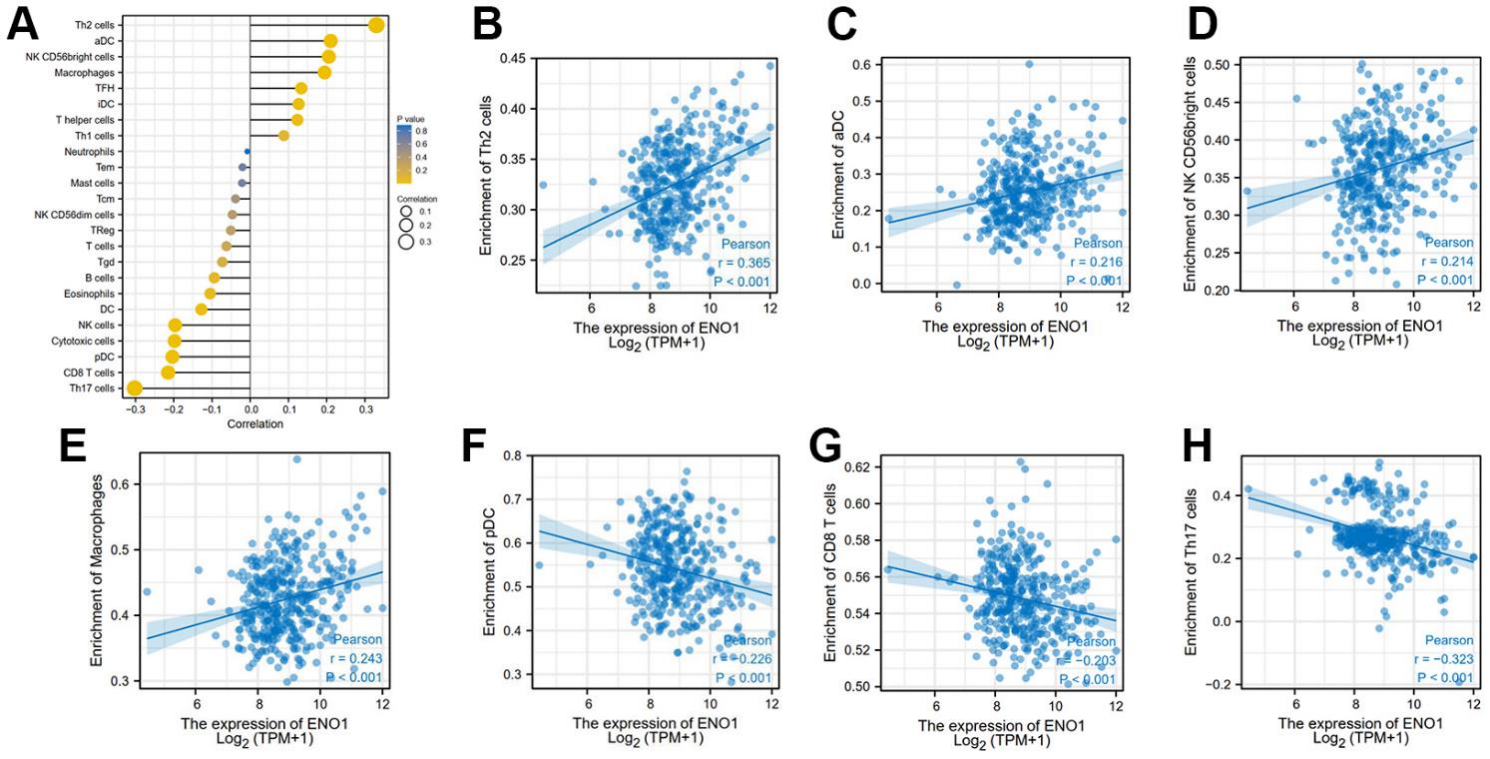
**High expression of hub gene ENO1 correlates with tumor immune infiltration.** The expression of ENO1 (**A**) correlated with Th2 cells (**B**), aDC (**C**), NK CD56bright cells (**D**), macrophages (**E**), pDC (**F**), CD8 T cells (**G**) and Th17 cells (**H**) in the tumor microenvironment.

The results of the clinical correlation analysis of ENO1 gene expression suggest that ENO1 is associated with tumor size and stage. The gene was less expressed in stage T1 compared to stage T2 and T3 (Figure 6A). The expression of ENO1 increased significantly with higher tumor stage in stage (Figure 6B), and in the pathological Grade stage, the expression of ENO1 was also increased in other Grade grades compared to Grade 1 (Figure 6C). However, unfortunately, our analysis revealed that ENO1 was not associated with lymph node status or distant metastatic status (Figure 6D, 6E). Furthermore, the immune infiltration results suggested that ENO1 correlated with Th2 cells, aDC, NK CD56bright cells, macrophages, pDC, CD8 T cells and Th17 cells in the tumor microenvironment, suggesting that ENO1 may promote liver cancer progression by regulating immune infiltrating cells in the tumor microenvironment (Figure 7).

## DISCUSSION

In this study, we screened seven gene biomarkers related to the prognosis of HCC from the perspective of oxidative stress, measured five targets expression (ENO1, NDRG1, NPM1, TXNRD1 and IL-33) of the seven gene biomarkers to investigate the reliability of the multi-index prediction in clinic, increasing the sensitivity and specificity of the predictive model and resulting in a significant increase in overall confidence.

Numerous previous studies have reported biomarkers associated with the prognosis of HCC. Liu et al. showed that the expression of PGM5 in HCC was significantly lower than that in adjacent tissues [15]. Wang et al. found that the expression of PPM1G gene in HCC tissues was lower than that in adjacent tissues [16]. However, the above studies were based on the evaluation of single-gene biomarkers, the clinical application was limited and there were also certain clinical biases. Therefore, it is particularly important to develop new polygenic models to predict the survival rate of HCC patients. Studies have reported that oxidative stress-related indicators are highly correlated with the prognosis of HCC patients. Huang et al. indicated that found that the level of glutathione in HCC tissues of patients was significantly higher than that in adjacent normal tissues [17]. Xiong et al. shown that PCK gene expression was down-regulated and was associated with poor prognosis in HCC patients [18]. Given the importance of oxidative stress, we tried to screen multi-gene biomarkers to predict the prognosis of liver cancer from the perspective of oxidative stress.

In this study, we performed Cox multiple regression analysis on the RNA sequencing data of HCC downloaded from the TCGA database, and identified the genes related to oxidative stress in patients. Among them, 7 genes met the selection criteria. We further analyzed related genes. Survival analysis showed that patients with high-risk scores had significantly shorter OS time compared with patients with low-risk scores. At the same time, risk score was a better predictor of patient survival than other specific medical parameters including age, tumor stage and histological type, suggesting these 7 genes deserve further study.

To more accurately predict patient 5- and 10-year survival, we developed a new nomogram in combination with other clinical factors. Compared with traditional classification systems for tumor nodules and metastases, or nomograms using only a single biomarker, the new nomogram we designed and developed may more accurately guide patients with poor survival rates to the correct treatment regimen. Among the seven genes identified, expression of ENO1, H2AX, MT3, NDRG1, NPM1 and TXNRD1 was risk-associated, suggesting that the expression levels of these genes are inversely correlated with HCC survival time. In contrast, the expression level of IL-33 was positively correlated with survival. ENO1, NDRG1 and NPM1 are reportedly associated with cancer as further discussed. We further measured the expression of ENO1, NDRG1, NPM1, TXNRD1 and IL-33 to investigate the reliability of the multi-index prediction. The data indicated that the oxidative stress-related polygenic biomarkers we screened are reliable and clinically significant in predicting prognosis.

As a hub gene of signature identified by PPI network, ENO1 is overexpressed in 70% of human cancers and is associated with poor cancer prognosis, converts 2-phosphoglycerate to phosphoenolpyruvate, plays an important role in the glycolytic pathway and the Warburg effect in cancer cells [19, 20]. High expression of ENO1 increased the risk score and the likelihood of poor prognosis, act as a biomarker in patients with hepatocellular carcinoma, and may be a favorable candidate for targeted treatment [21]. NDRG1, one member of NDR- and an α/β hydrolase-fold region family [22]. Both NDRG1 and ENO1 are closely related to glycolysis and carcinogenesis. Studies have shown that NDRG1 play important roles in tumor invasion and metastasis [23]. Compared to healthy controls, NDRG1 expression is upregulated in HCC patients and is associated with poorer prognosis and histological grade [24, 25]. NPM1, abundant nucleolar proteins that often shuttle between the nucleolus and the nucleoplasm or cytoplasm, is involved in chromatin remodeling, genome stabilization, cell cycle progression, and apoptosis in cancer [26]. The study showed that liver cancer patients with lower levels of NPM1 have a better prognosis [27]. MT3 protects the body from DNA damage, and H2AX is a biomarker of DNA damage [28, 29]. As one of the major REDOX regulators, TXNRD1 is associated with tumor aggressiveness and poor prognosis [30]. Patients with high expression of IL-33 in their tumors had higher survival rates. These indicate the importance of these 7 indicators in tumor prognosis.

Although we have used multigene biomarker analysis and the characteristics of the 7 genes screened can effectively predict the prognosis of HCC patients, this study still has certain limitations. Future studies are needed to improve the accuracy of the risk scoring model with additional patient cohorts. In addition, a patient’s treatment regimen is critical to cancer prognosis, incorporating patient treatment regimen data into these analyses will add value to subsequent results.

## CONCLUSIONS

The seven gene signatures established in this study are effective and stable in HCC samples from TCGA. These 7 gene signatures are autonomous factors for the prognosis of HCC patients. These results may help develop more effective prognostic tools and ultimately improve patient outcomes.

## MATERIALS AND METHODS

### Data acquisition and pre-processing

Data from sequenced liver cancer samples were obtained from the public database TCGA, a dataset containing a total of 50 paraneoplastic and 371 cancerous tissues. Clinical information matching the patient samples was likewise downloaded. Patients with less than 30 days of follow-up were also removed based on survival information. Oxidative stress-related genes were obtained from the gene card database and these genes have been confirmed by previous experiments.

### Differential gene analysis

We extracted oxidation-related gene expression profiles from transcriptomic data based on information from screened clinical samples and performed differential analysis using the limma package, differential genes were defined as those genes whose gene expression in cancer and paracancer met |LogFC| > 1 while p < 0.05. FC: Fold change.

### Univariate Cox regression and LASSO regression

Univariate Cox regression was used to filter prognostic genes associated with patients’ overall survival (OS), and genes that met the criteria were further entered into the LASSO regression model for selection of significant genes among these genes, which were done by the survival package and the caret package respectively. A p-value less than 0.05 was defined as statistically significant in the univariate Cox regression. The best model variable was obtained by taking the minimum value of Lambda in the LASSO regression. The lambda.min is the optimal value of λ found during the cross-validation process, used to minimize prediction error, rather than just being the smallest possible value of λ.

### Multi-Cox regression and prognostic model construction

The results of the above analysis were further incorporated into a multifactorial Cox regression model to identify independent prognostic genes, a procedure performed by the survival package. Finally, we constructed a multi-gene prognostic model based on the coefficients of this regression model and the expression profile of the genes. Based on the scores of the model, we stratified patients and compared survival differences between groups, using ROC to assess the predictive efficacy of the model. To test the robustness of the model, we also randomly extracted 30% of the data for validation.

### Prognostic model with tumor detection

Back propagation (BP) network was used to assess the ability of the model genes to detect early-stage liver cancer. We first transformed the raw count into TPM data and calculated the genescore of the model genes based on the results of the difference analysis and the expression of the model genes in the genescore matrix, where genescore in the genescore matrix, genescore greater than the median expression value is defined as 1, and those less than the median expression value is defined as 0. Finally, we input the genescore matrix into the BP network and visualise it, and the area under the ROC curve is used to assess the predictive power.

### PPI identify hub gene of model and validate expression

We performed PPI protein interaction network analysis on the genes in the model through an online database, STRING database, and defined the hub genes with multiple gene links as core genes. We further validated the expression of the core genes at the transcriptional and post-transcriptional levels.

### Hub gene with clinical factors and immune infiltration

To further assess the clinical value of the core gene, we correlated the core gene with common clinical variables (T, N, M, stage, grade and tumor status). We removed samples missing the above variables from the original clinical samples and looked at the differences in expression of hub gene between the variable groups. ssGSEA was used to assess the relationship between hub gene expression and immune infiltration cells in the tumor microenvironment.

### Clinical information of the patients

20 patients with HCC confirmed by histopathology who received routine surgical resection at The First Affiliated Hospital of Nanjing Medical University from 2022 to 2023 were included in this study. The inclusion criteria for patients (all three items needed to be met simultaneously) were as follows: 1) patho-logically confirmed HCC; 2) no preoperative surgery, chemotherapy or radiotherapy for tumours; and 3) complete matched tumour and adjacent nontumour paraffin-embedded specimens. These patients included 16 males and 4 females, ranging in age from 43 to 74 years, and the median age was 59 years.

The clinicopathological information of all patients, including tumour size, tumour number, Edmondson and Steiner system of HCC (ES) grading, and Micro vascular (MVI) invasion, was collected. The use of tissue specimens in this study was approved by the Ethics Committee of the First Affiliated Hospital of Nanjing Medical University before the start of the study.

The essential clinical information of the patients is presented in Supplementary Table 4.

### Immunohistochemical analysis

The antibody including against ENO1 (1:100, 11204-1-AP, Proteintech, CA, USA), NDGR1 (1:100, 26902-1-AP, Proteintech, CA, USA) and NPM1 (1:100, 60096-1-AP, Proteintech, CA, USA) TXNRD1 (1:100, 11117-1-AP, Proteintech, CA, USA), IL-33 (1:100, 123726-1-AP, Proteintech, CA, USA) were incubated at room temperature for 2 h. Subsequently, the secondary antibody was used in incubating the slice at 37° C for 30 min, more details were shown in the Supplementary Materials.

### Histological analysis

Samples were fixed in 4% paraformaldehyde (PFA) at 4° C overnight. Samples were processed using conventional techniques. 5 μm sections were cut and stained with haematoxylin and eosin (H&E) to observe the morphology of the tissue.

### Data availability

The datasets were obtained from the TCGA databases. Oxidative Stress (OS) related genes were checked by the Gencard database (https://www.genecards.org/). The datasets during and/or analyzed during the current study are available from the corresponding author on reasonable request.

## Supplementary Material

Supplementary Materials

Supplementary Table 1

Supplementary Table 2

Supplementary Table 3

Supplementary Table 4
