# Supplementary material for: Seven oxidative stress-related genes predict the prognosis of hepatocellular carcinoma
Source: Aging (Albany NY). 2023 Dec 14;15(24):15050–63. doi: 10.18632/aging.205330 (PMC10781471; doi:10.18632/aging.205330)
Supplement: Supplementary Materials [file aging-15-205330-s001.pdf]

SUPPLEMENTARY MATERIALS

Immunohistochemical procedure

Paraffin-embedded, formalin-fixed HCC and adjacent non-tumor tissue slices were deparaffinized and rehydrated, then the slices were pre-treated using pressure cooker heat mediated antigen retrieval with sodium citrate buffer (pH=6) for 12 mins. Endogenous peroxidase activity was blocked by incubation with 3% hydrogen peroxide for 10 min. After washing with phosphate buffered saline (PBS) 3 times, the corresponding specific primary antibodies were applied to stain each slice overnight at 4° C. The antibody includes against ENO1 (1:100, 11204-1-AP, Proteintech, CA, USA), NDGR1 (1:100, 26902-1-AP, Proteintech, CA, USA) and NPM1 (1:100, 60096-1-AP, Proteintech, CA, USA) TXNRD1 (1:100, 11117-1-AP, Proteintech, CA, USA) IL-33 (1:100, 123726-1-AP, Proteintech, CA, USA). Subsequently, the secondary antibody was used in incubating the slice at 37° C for 30 min. We then used diaminobenzidine (DAB) solution (Dako, Denmark) to stain the slices, and the slices were counterstained with haematoxylin. Two experienced pathologists, who had no prior knowledge of patient clinical data, independently assessed the IHC staining.

Evaluation of IHC-positively stained tissues

The H-score method was applied for calculating the staining score of each sample, which was to multiply the immunoreaction intensity (negative: 0, weak: 1, moderate: 2, strong: 3) by the staining extent score (0%-100%). According to the H-score, the stained samples were divided into four groups: negative (-; 0), weak (+; 0~1), moderate (++; 1~1.5), and strong (+++; 1.5~3). Samples with a negative or weak H-score were determined to be the low protein expression group, whereas those with a moderate or strong H-score were classified as the high protein expression group.
