# Supplementary material for: Seven oxidative stress-related genes predict the prognosis of hepatocellular carcinoma
Source: Aging (Albany NY). 2023 Dec 14;15(24):15050–63. doi: 10.18632/aging.205330 (PMC10781471; doi:10.18632/aging.205330)
Supplement: Supplementary Table 4 [file aging-15-205330-s005.pdf]

Supplementary Table 4. Patients' essential characteristics.

Characteristic		n
Age (years)	<55	6
	≥55	14
Gender	Male	16
	Female	4
tumor size	≤5	7
	>5	8
tumor number	1	18
	>1	2
ES	I~II	10
	II~IV	10
MVI	0	10
	1-2	10
